# 

**Published:** 1801-08

**Authors:** 


### To CORRESPONDENTS.

*Those Lecturers who wish their Autumnal Courses to be announced in the next number, are desired to send their Notice by the 15th of August.*

